# Out Door Life for Women

**Published:** 1885-06

**Authors:** 


### Out-Door Life for Women.

The redemption of women's health, I am more and more convinced, depends on their taking to out-door life and activities. Reading high class memoirs which are in everyone's hands now-a-days, of the Carlyles, the Sterlings and F. D. Maurice, one is distressed to hear the continual story of weak health, and women who, brought to face the realities and efforts of life, immediately droop, languish, and are a long time dying. If they have a house to keep, and a share of the actual work, like Mrs. Carlyle, at Craigenputtock and Chelsea, they sicken mysteriously, and their life is a time of wrestling with househould affairs, alternating with refuge on the sofa, or months in the doctor's hands, in that wretched, unimprovable state which justified the sigh of a much tried husband who "wished his wife would get better, or something!" Have I not, through the ignorance of my day and generation, wasted life enough in attacks of the familiar household demon, nervous prostration, which only vanishes on turning the patient out of doors. Twice and again, friends have looked pityingly on me as good as gone, but taken out of doors ten hours a day, as good for nothing else, sun and wind wrought their spell of healing, and health came again. Henceforth no more in-door life than must be for me, and I would urge other women to fashion their lives so as to spend them more in the open air.—From "How to Dress for the Garden," in *Vick's Magazine* for May.

Rose Bugs.—It is said that Paris green applied to rose bushes and grape vines infested with rose bugs will kill the insects as surely as it does the potato bug, when used on potato plants. The application can be dry, mixed with flour, or land plaster, or in liquid form, mixed with water, and sprinkled on, in the same manner as for the potato bug.—*Vick's Magizine* for June.

